# Editorial: Challenges and strategies in the management of ER/PgR Low-expression breast cancer: exploring fundamentals, clinical insights, and treatment approaches

**DOI:** 10.3389/fonc.2026.1878814

**Published:** 2026-06-11

**Authors:** Takashi Takeshita, Vittorio Gebbia, San-Gang Wu

**Affiliations:** 1 Department of Breast and Endocrine Surgery, Kumamoto City Hospital, Kumamoto, Japan; 2 Chair of Medical Oncology, Kore University of Enna, Enna, Italy; 3 First Affiliated Hospital of Xiamen University, Xiamen, China

**Keywords:** estrogen receptor (ER) low expression, hormone receptor-low breast cancer, immunohistochemistry (IHC), Triple-negative breast cancer (TNBC), tumor microenvironment

Breast cancer management is fundamentally shaped by hormone receptor (HR) status, yet tumors with low immunohistochemical (IHC) expression of estrogen receptor (ER) or progesterone receptor (PgR)—broadly defined as 1–10% nuclear staining—occupy a contested biological and therapeutic territory between luminal and triple-negative breast cancer (TNBC). The current ASCO/CAP 1% positivity threshold does not adequately capture the heterogeneity of this subgroup, leaving clinicians uncertain about the optimal roles of endocrine therapy, CDK4/6 inhibitors, chemotherapy, and emerging agents. This Research Topic assembled seven articles spanning biomarker biology, adjuvant therapy, systemic treatment, novel drug delivery, surgical innovation, and atypical clinical presentations, providing a multidisciplinary portrait of an underserved patient population.

A unifying molecular framework for this Research Topic is provided by the editors’ own work, Dual Cutoffs of Estrogen Receptor Positivity Define Prognostic and Predictive Subgroups in Breast Cancer (Takeshita et al., 2026). Integrating IHC-based ER quantification with transcriptomic and immune profiling across four HER2-negative cohorts, this study identified ER expression as a continuous prognostic variable, with recurrence risk peaking near 45% and declining beyond 50%. Tumors with ER ≥50% exhibited favorable survival and immune-depleted, luminal signatures, whereas ER < 50% tumors—including ER-low cases—showed TNBC-like proliferative, metabolic, and p53/DNA-repair pathway activation alongside enriched B-cell and macrophage infiltration. These dual cutoffs (1% and 50%) offer a biologically grounded reclassification that directly contextualizes the therapeutic uncertainties described throughout this Topic.

The prognostic interplay between low PgR, high Ki67, and HER2 overexpression is examined at the pre-invasive stage by High Ki67 expression, HER2 overexpression, and low progesterone receptor levels in high-grade DCIS: significant associations with clinical practice implications (Schandiz et al.). In 484 DCIS cases, low PgR combined with high Ki67 was significantly associated with HER2 overexpression (p = 0.0107), with only moderate interobserver agreement for Ki67 counting (κ = 0.586), underscoring the need for standardized biomarker assessment. These findings suggest that aggressive biomarker profiles originating in DCIS may foreshadow the molecular characteristics of invasive ER/PgR-low disease.

The most clinically pressing question—whether ER-low patients benefit from adjuvant CDK4/6 inhibitors and endocrine therapy—is rigorously examined by Adjuvant endocrine therapy in estrogen-low breast cancer in the era of CDKI: are we at another uncertain crossroads? (Valerio et al.). This review synthesizes MONARCH E and NATALEE exploratory subgroup data alongside molecular profiling, concluding that approximately 75–80% of ER-low tumors are non-luminal and basal-like, with outcomes closer to TNBC than ER-high disease. Numerically consistent, though not statistically significant, CDK4/6 inhibitor benefit appears mediated by ER-independent RB-pathway and immunomodulatory mechanisms rather than classical endocrine signaling. Palbociclib-based trials showed no benefit, attributed to pharmacological class differences. Molecular subtyping (PAM50, RB1, PR, germline BRCA) before adjuvant CDK4/6 inhibitor use and dedicated prospective trials with ER-low as a pre-specified stratum are urgently recommended.

Two articles address chemotherapy and immunotherapy in HER2-negative/TNBC disease—categories that substantially overlap with the ER-low subgroup. Efficacy and safety of Eribulin-based chemotherapy in HER2 negative advanced breast cancer patients: a real-world study (Li et al.) demonstrated that earlier-line eribulin use in 105 Chinese patients yielded significantly longer progression-free survival (PFS: 6.7 vs. 4.9 months; p = 0.038), and that combinations with PD-1 inhibitors outperformed anti-angiogenic combinations (p = 0.022). Complementarily, PD-1/PD-L1 inhibitors plus chemotherapy versus chemotherapy alone for Asian patients with advanced triple-negative breast cancer: a phase III RCTs based meta-analysis (Ruan et al.) confirmed in 1,041 Asian TNBC patients that PD-1/PD-L1 inhibitor plus chemotherapy significantly improved PFS (HR 0.74; p = 0.0008) and, among PD-L1-positive patients, overall survival (HR 0.62; p = 0.005). Given the immune-enriched microenvironment of ER-low tumors documented by our profiling, these results support prospective evaluation of checkpoint inhibitors in ER/PgR-low disease.

Novel drug delivery strategies are explored by Nanoemulsion of myricetin enhances its anti-tumor activity in nude mice of triple-negative breast cancer xenografts (Sharma et al.), in which a myricetin nanoemulsion (Myr-NE) significantly outperformed myricetin alone in TNBC xenografts by inhibiting PI3K/AKT/mTOR signaling and VEGFR2, reducing Ki67 and MMP9, and inducing greater oxidative stress. Given the PI3K/AKT/mTOR pathway activation identified in ER-low tumors by transcriptomic profiling, phytochemical nanomedicine represents a promising investigational avenue.

Surgical quality-of-life considerations are addressed by Clinical outcomes and aesthetic results of reverse sequence endoscopic versus traditional bilateral nipple-sparing mastectomy with immediate implant-based breast reconstruction (Zhang et al.). In 116 patients, the novel reverse-sequence endoscopic technique achieved lower complication rates (6.6% vs. 32.5%; p < 0.001) and superior cosmetic scores (Scar-Q: 81.32 vs. 35.17; p < 0.001) compared with conventional open bilateral reconstruction, without prolonging operative time.

The diagnostic challenges of ER/PgR-low disease are vividly illustrated by Extensive thoracic vertebral and chest wall metastases as the initial presentation of breast cancer: a case report and literature review (Kenzhegulov et al.), in which widespread thoracic skeletal metastases from an occult breast primary initially mimicked multiple myeloma, highlighting the need for high oncologic suspicion, PET-CT, and histological verification in patients presenting with atypical or undifferentiated tumors.

Collectively, these contributions confirm that ER/PgR low-expression breast cancer cannot be adequately managed through the lens of conventional binary HR classification. A biologically grounded, expression-level–informed approach—integrating molecular subtyping, immune profiling, and evidence-based selection of endocrine therapy, CDK4/6 inhibitors, chemotherapy, or immunotherapy—is essential for improving outcomes. Dedicated prospective trials enrolling ER-low patients as a distinct stratum are an urgent priority. We thank all contributing authors, reviewers, and the editorial office of *Frontiers in Oncology* for their support in advancing this important field.

